# The Parkinson Care Advocate: Integrating Care Delivery

**DOI:** 10.3389/fneur.2017.00364

**Published:** 2017-07-27

**Authors:** Leonard L. Sokol, Debbie Shapiro, Michael J. Young, Adina H. Wise, Uri P. Hadelsberg, Yakir Kaufman, Alberto J. Espay, Aristide Merola

**Affiliations:** ^1^University of Cincinnati College of Medicine, Cincinnati, OH, United States; ^2^Department of Neurology, James J and Joan A. Gardner Center for Parkinson’s Disease and Movement Disorders, University of Cincinnati, Cincinnati, OH, United States; ^3^Tikvah for Parkinson, Jerusalem, Israel; ^4^Departments of Internal Medicine and Neurology, Massachusetts General Hospital, Boston, MA, United States; ^5^Harvard Medical School, Boston, MA, United States; ^6^Columbia University Medical Center, New York, NY, United States; ^7^Department of Neurosurgery, Shaare Zedek Medical Center, Jerusalem, Israel; ^8^Department of Neuropsychogeriatrics, Herzog Hospital, Jerusalem, Israel; ^9^Faculty of Medicine, Hebrew University, Hadassah Medical Center, Jerusalem, Israel

**Keywords:** Parkinson, patient-centered, public health, secondary prevention, cost–benefit

## An Increasing Problem with Multiple Challenges: Parkinson’s Disease (PD)

With a prevalence of 1% in the population older than 65 years old, PD is recognized as the second most common neurodegenerative disorder after Alzheimer disease. PD affects approximately eight million people worldwide (1), more than the combined number of patients diagnosed with multiple sclerosis, muscular dystrophy, and amyotrophic lateral sclerosis (2).

Clinically, PD represents a complex and multifaceted syndrome characterized by a variable combination of motor and non-motor symptoms (3). Motor symptoms include tremor, rigidity, and bradykinesia, frequently associated with alteration of postural stability. Non-motor symptoms include cognitive dysfunctions (frontal dysexecutive syndrome, eventually resulting in cognitive impairment), mood–behavioral disorders (impulsivity, anxiety, and depression), cardiovascular alterations (orthostatic hypotension and supine hypertension), fatigue, sleep abnormalities, and gastrointestinal and urinary dysfunctions.

For over two decades, care for chronic, neurological disorders, including PD, has been fragmented (4). Lack of integration in care delivery has potentiated numerous misconceptions among patients and providers (5, 6), including erroneous understandings of the natural course of PD and of the availability and utility of various treatment modalities (6–8). In addition, blossoming time constraints in many settings frequently limit the possibility to address the crucial roles of physical therapy, dietary therapy (9–12), and fall-prevention programs which can reduce the morbidity and the cost burden of the disease (13) and often thwart the first cause of hospitalization for PD patients.

## A Parkinson Care Advocate (PCA) to Promote Continuity of Care

According to Freeman and colleagues’ notion of continuity of care (4), several elements converge to promote the highest quality of care, including relationships, management, information, societal context, and personal agency (volition). Initial qualitative exploration (14) that elicited desires from PD patients and caregivers demonstrated alignment with Freeman’s attributes. Patients’ articulated desires to receive assistance with diagnostic acceptance and prognostication; to obtain accurate information surrounding the disease; and to experience integrated care and periodic follow-up as modalities of treatment evolve (14). As in other neurodegenerative diseases, the therapeutic importance of fortifying a patient’s sense of agency in a phenomenological context that powerfully challenges the sense of self is immense (15, 16).

**Figure 1:**
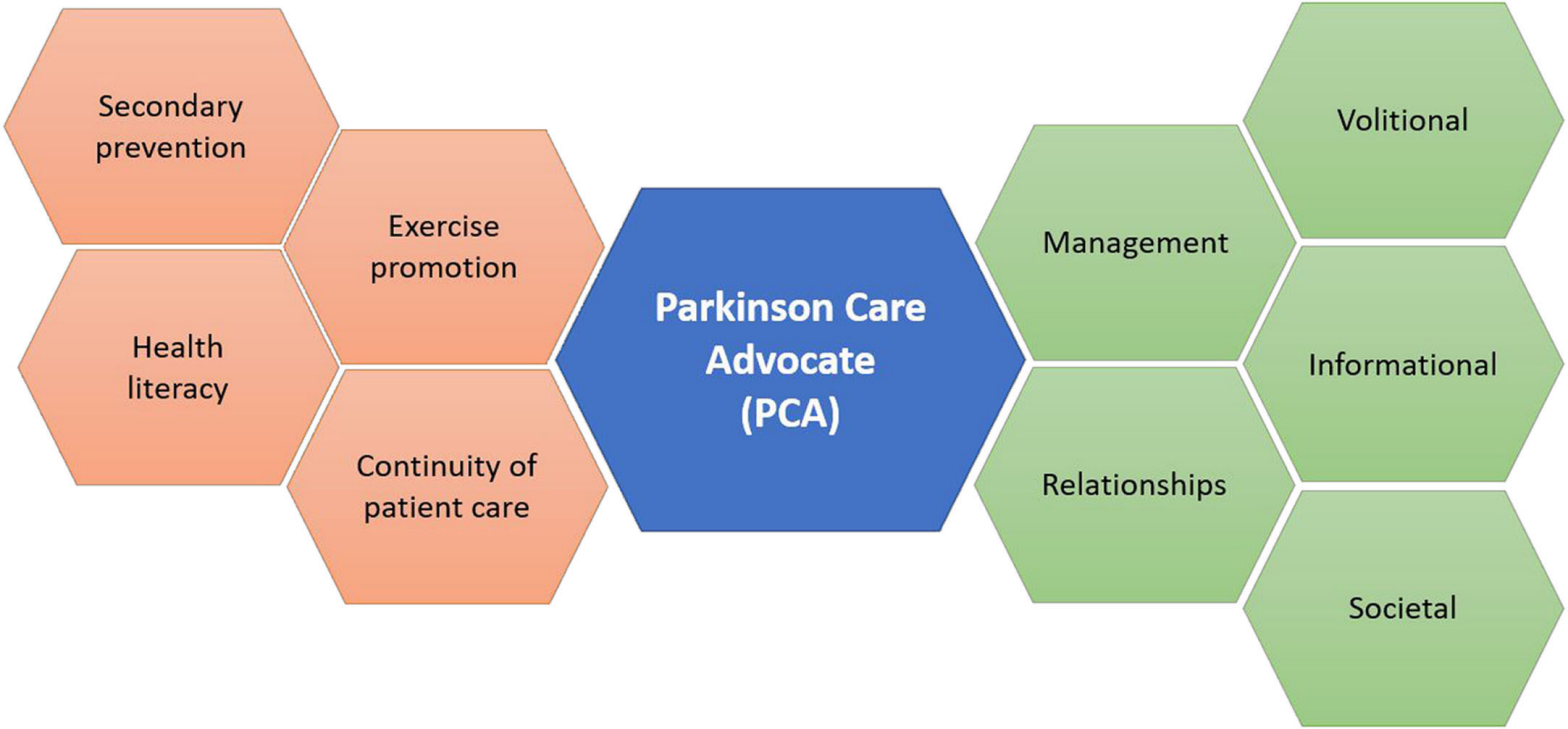
The Parkinson Care Advocate (PCA) will serve to integrate and coordinate multiple dimensions of Parkinson’s disease care, in tune with themes from Freeman’s model.

We, therefore, hypothesize that embodiment of Freeman’s continuity of care model through development and implementation of the PCA may decrease the prevalence of misconceptions about PD among patients and their family members (Figure 1) in addition to promoting coordination and integration of PD care delivery. Similar to the ParkinsonNet (17, 18) model, which involved standardization of physiotherapy for PD patients (17), this model emphasizes that PCAs undertake integrative (19, 20), educational roles within specific key cohorts, including those with poor treatment compliance, low health literacy (21), or advanced-stage disease. Akin to the diabetes educator (22) and other educational providers, implementation of PCAs portend improvements in clinical outcomes through fostering continuity of patient care, surmounting barriers in health literacy, coordinating tailored exercise sessions, and promoting cost-effective programs targeted at prevention (23).

## Education—Internet, Groups, and Health Literacy

Disease education is vital to treatment success (11, 24). PD patients obtain knowledge about their condition from their neurologists (20), whose abilities to engage in comprehensive disease counseling may be limited by time and resource constraints (4); from non-specialized consultants who patients may sparingly visit (13); and from the Internet, often a source of misinformation (24). Regardless of modality, acquiring accurate and actionable information about the disease is crucial. This is especially pressing among patients with reduced health literacy (24) who may experience higher rates of medical non-adherence as a result (24). Community-based studies found that patients feel that inadequate time with their health-care teams is devoted to education (4, 23). While a multitude of Internet resources exist, past research (24) has suggested that only 30% of those over 60 years old use the Internet for health-related information. Moreover, much of the information that may be encountered online may be inaccurate, vague, or outdated (25–27).

If PD patients do seek information via the Internet (a 2017 Google search for “Parkinson’s disease” returned approximately 15,400,000 results), is the available information evidence-based and comprehensible to those who require it (7)? The US Department of Health and Human Services (USDHHS) recommends that health literature be composed at a reading level between the United States equivalent of fourth- and sixth- grade levels (24). This recommendation is at odds with a recent assessment (24) of the top 100 PD web-pages returned from a search, which revealed that most consumer-focused PD web-pages were written at a college undergraduate level, with only 0–4% of these pages satisfying the USDHHS recommendations. Although this specific disease group’s literacy rates have not been empirically explored, PD is primarily a disease of the elderly (aged 65 and older), 61% of whom have a basic or below basic-reading level (24).

To surmount these barriers to comprehensive PD education, initial evidence suggests a role for a PCA-like provider in group (6, 28) and individual (23) settings. For newly diagnosed patients (*n* = 24) and their caregivers, a 3-h educational session provided information and psychological support (6). Around 87% of respondents believed the session contributed to their ability to explain their illnesses to family and friends; 68% stated that the session aided in their acquaintance with staff; and 78% replied that it made them feel welcome.

Of those with low-health literacy, many will not have achieved a college-level education and will have limited command of the English language. PCAs will, therefore, be charged with translating and formatting written and oral materials into the appropriate dialect at the appropriate literacy level in accordance with USDHHS recommendations. Tailoring the delivery of this knowledge to its intended audience will increase comfort among PD patients with their diagnosis and may reduce the incidence of hospitalizations which are secondary to non-adherence vis-à-vis poor education. Third, as caregivers and family members are often neglected during disease planning, an active and involved PCA may prove salutary for the social, psychological, spiritual (29, 30), and physical well-being of patients’ caregivers and family members (31).

## Tailored Care through Exercise

Several mechanisms (9) explain the motor- and non-motor- benefits PD patients derive from various types of exercise (Table 1), including but not limited to the prevention of secondary complications, such as falls (13, 32). This notion might be even more salient within certain ethnicities, as data, for example, suggest that PD-carriers for pathogenic variants in *LRRK2* or *GBA* portend greater risk for freezing of gait and a higher risk for falls (33). Customized plans, through PCAs, thus, should be designed (10), that are personalized to both disease severity (5) and PD phenotype, including postural instability and cognitive dysfunction (33).

**Table 1:** Mechanisms and types of exercise^a^ for Parkinson’s disease (PD) (9).

Mechanism	Type	Benefit
Neuroplasticity^b^ Neuroprotective^c^ Neurorestorative^d^	Goal-based^e^ Treadmill training Amplitude training Tai Chai Tango dancing Boxing	Improvement of gait velocity Improvement in Unified Parkinson disease Rating Scale score Reduction in stride length variability Restoration of automaticity Cognitive improvements Sleep improvements Promotion of self-efficacy
	Goal-based^e^ and aerobic^f^ Treadmills Cycling	

*^a^Defined as willed, repetitive movements that strive for a given goal*.

*^b^Mechanism contends either alterations in synaptic transmission, perhaps reflective of differential expression of dopaminergic transporters or of synaptic morphology, whereby dendritic spine loss is attenuated from the medium, spiny neurons found within the basal ganglia’s inhibitory circuit*.

*^c^Mechanism contends a halting of the elusive neurodegenerative cascades*.

*^d^Non-specific (general) effects include neurogenesis in medial temporal regions; release of angiogenic and anti-inflammatory factors (e.g., VEGF, HIF, IL-10), modification of the myeloid-associated population implicated in pathogenesis of PD; and diminution of gliosis within subcortical structures*.

*^e^Group of exercises that result in improvements in features, such as gait and balance*.

*^f^Group of exercises that tax the cardiopulmonary system and may improve automaticity through rejuvenation of the striato–cortico–thalamic circuit*.

Under the supervision of physical therapists and neurologists, PCAs might be charged with coordinating exercise regimens during the most opportune times, consistent with the ON-therapeutic window [“flexibility continuity” (4)] but also with set days and times [“longitudinal continuity” (4)]. Additionally, with the advent of PD wearables that may inform providers of functional status (34), PCAs might serve as human adjuncts [“relational continuity” (4)], sensitive to non-motor features undetected by wearables and promoting the early activation of programs to preserve mobility.

Results from a 2016 study (13) from 231 Australian PD patients, evaluating the cost-effectiveness of a 6-month secondary prevention program for PD patients, found that fewer participants in the intervention (exercise), as compared to the control (no exercise) group, experienced declines in mobility, and the intervention saved 574 AUD and 9,570 AUD for each fall prevented and for each participant who staved off further deterioration in mobility, respectively. Overall, the intervention yielded an 80% chance of cost-effectiveness and participants in the intervention group demonstrated marked economic benefits among all clinical measures, including total falls, frequency of those avoiding severe impairments in mobility, and quality of life years.

## Conclusion

Parkinson care advocates will follow their patient base continuously, in tune with Freeman’s model of care. Basic knowledge of PD personalized to patients’ literacies will be provided and community resources offered *via* meetings within the home or group settings. Exercise regimens aimed at secondary prevention can also be planned under appropriate guidance. The PCA can foreseeably serve as a central point of contact to assess and relay progress to the treatment team, answer questions, coordinates referrals, and offer encouragement. The program’s cost-effectiveness foreseeably offsets investment costs through maintaining or improving QoL, preventing secondary complications, and delaying the need for skilled nursing facility placement. To these ends, this initiative will be instrumental in promoting a more comprehensive, patient-centered, and cost-effective approach to Parkinson care.

## Author Contributions

LS conceived of the idea, wrote the first draft of the manuscript, and revised subsequent drafts for intellectual content. DS conceived of the idea and revised the manuscript for intellectual content. MY added and revised the manuscript for important intellectual content. AW added and revised the manuscript for important intellectual content. UH contributed to the intellectual concepts and added and revised the manuscript for important intellectual content. YK, AE, and AM added and revised the manuscript for important intellectual content.

## Conflict of Interest Statement

LS has served on the executive editorial board for Carnegie Mellon University’s Triple Helix Journal for Science, Society, and Law, and has received remuneration from Yahoo!, Johnson & Johnson, and Tablet Magazine. He is an *ad hoc* consultant for Tikvah for Parkinson. DS is founder of Tikvah for Parkinson. She has served as managing editor for BreslovWorld. She has received publishing royalties from Feldheim; Breslev Research Institute; Israel Book Shop; Yafeh Nof; Jerusalem Publications; Art Scroll Publishers; http://Aish.com; Bina; http://www.breslev.co.il; http://Chabad.org; Hamodia; Horizons; Inspiraion; Jerusalem Post; Jerusalem Report; Jewish Homemaker; Jewish Lifestyles; Jewish Observer; Lakewood Shopper; http://OU.org; Voice of Lakewood; Yated Ne’eman American Edition; Yated Ne’eman Israeli Edition; and YeshivaWorld News. MY and AW have nothing to disclose. UH is an *ad hoc* consultant for Tikvah for Parkinson. YK is a medical advisor to Tikvah for Parkinson. AE has received grant support from NIH, Great Lakes Neurotechnologies, and the Michael J Fox Foundation; personal compensation as a consultant/scientific advisory board member for Abbvie, TEVA, Impax, Merz, Acadia, Cynapsus, Lundbeck, and USWorldMeds; publishing royalties from Lippincott Williams & Wilkins, Cambridge University Press, and Springer; and honoraria from Abbvie, UCB, USWorldMeds, Lundbeck, Acadia, the American Academy of Neurology, and the Movement Disorders Society. He serves as Associate Editor of the Journal of Clinical Movement Disorders and on the editorial board of Parkinsonism and Related Disorders. AM has received grant support from UCB Pharma and speaker honoraria from CSL Behring, UCB Pharma and Teva Pharmaceuticals. He has received personal compensation from Edge Consulting S.r.l., MediK S.r.l., and Sthetos S.r.l.

## Abbreviations

PD, Parkinson’s disease; PCA, Parkinson care advocate; QoL, quality of life; USDHHS, US Department and Health and Human Services; UPDRS, Unified Parkinson disease Rating Scale; PT, physical therapist.
